# Hierarchical Fabrication of Plasmonic Superlattice Membrane by Aspect-Ratio Controllable Nanobricks for Label-Free Protein Detection

**DOI:** 10.3389/fchem.2020.00307

**Published:** 2020-04-28

**Authors:** Yi Chen, Huang Liu, Haojing Yin, Qi Zhu, Gang Yao, Ning Gu

**Affiliations:** ^1^State Key Laboratory of Bioelectronics, Jiangsu Key Laboratory for Biomaterials and Devices, School of Biological Science and Medical Engineering, Southeast University, Nanjing, China; ^2^Southeast University-Monash University Joint Research Institute, Suzhou, China; ^3^School of Pharmaceutical and Chemical Engineering, Chengxian College, Southeast University, Nanjing, China

**Keywords:** plasmonic, superlattice membrane, aspect-ratio controllable synthesis, SERS, label-free detection

## Abstract

Plasmonic superlattice membrane exhibits remarkable functional properties that are emerging from engineered assemblies of well-defined “meta-atoms,” which is featured as a conceptual new category of two-dimensional optical metamaterials. The ability to build plasmonic membranes over macroscopic surfaces but with nanoscale ordering is crucial for systematically controlling the light-matter interactions and represents considerable advances for the bottom-up fabrication of soft optoelectronic devices and circuits. Through rational design, novel nanocrystals, and by engineering the packing orders, the hybridized plasmon signature can be customized, promoting controllable near-field confinement for surface-enhanced Raman scattering (SERS) based detection. However, building such 2D architectures has proven to be remarkably challenging due to the complicated interparticle forces and multiscale interactions during self-assembly. Here, we report on the fabrication of ultralong-nanobrick-based giant plasmonic superlattice membranes as high-performance SERS substrates for ultrasensitive and label-free protein detection. Using aspect-ratio controllable short-to-ultralong nanobricks as building blocks, we construct three distinctive plasmonic membranes by polymer-ligand-based strategy in drying-mediated self-assembly at the air/water interfaces. The plasmonic membranes exhibit monolayered morphology with nanoscale assembled ordering but macroscopic lateral dimensions, inducing enhanced near-field confinement and uniform hot-spot distribution. By choosing 4-aminothiophenol and bovine serum albumin (BSA) as a model analyte, we establish an ultrasensitive assay for label-free SERS detection. The detection limit of BSA can reach 15 nM, and the enhancement factor reached 4.3 × 10^5^, enabling a promising avenue for its clinical application in ultrasensitive biodiagnostics.

## Introduction

The significant breakthroughs in multi-scale revolutionary technology enable both the innovation of nanoarchitectures and the expansion of macroscale functionalities (Zheludev and Kivshar, 2012; Laramy et al., 2019). Noble metallic nanoparticles exhibit programmable morphology and unique optoelectronic properties (Xia et al., 2009), such as localized surface plasmon resonance (LSPR) (Halas et al., 2011), plasmonic coupling and hybridization (Prodan et al., 2003), etc. They can be used as building blocks for the construction of nanocrystal-based superlattice materials (Tan et al., 2011), inducing great practical significance for flexible optoelectronic devices, biomedical sensing, cancer theranostics, and ultrasensitive biomarker detection (Wu et al., 2018). However, it has been notoriously challenging to efficiently assemble such “artificial metallic atoms” (Tan et al., 2011) into desired arrangements with highly ordered nanoscopic structures and distinctive multi-functionality.

Among the recently developed superlattice materials (Gong et al., 2012; Liu et al., 2016; Lin et al., 2018), the plasmonic superlattice membrane represents a new-generation of the thinnest possible two-dimensional metamaterials. Such a monolayered superlattice membrane consists of self-assembled metallic nanocrystals with closely packed nanoscopic structures and programmable multifunctions (Mueggenburg et al., 2007; Tao et al., 2007; Pang et al., 2008; Cheng et al., 2009; Dong et al., 2010, 2011; Chen et al., 2011, 2013; Liao et al., 2011), rendering a unique optical signature for considerable scope of applications such as nanoelectronics (Si et al., 2019), attachable SERS substrate (Chen et al., 2015), chiral sensors (Wu et al., 2018), to name a few. Since the concept of a “particle superlattice” was first introduced by Kotov et al. (1994), substantial top-down and bottom-up strategies have been developed to build plasmonic superlattices, including the Langmuir-Blodgett technique (Tao et al., 2007), the droplet evaporation method (Mueggenburg et al., 2007; Chen et al., 2011), interface-based assembly (Pang et al., 2008; Liao et al., 2011), and acoustic levitation technique (Shi et al., 2019a). For all those assembly strategies, soft ligands that capped around nanoparticles play critical roles in the regulation of nanoscale forces and interparticle potential, enabling ligand-engineering strategy to fine-tune the interparticle spacing and program superlattice properties (Si et al., 2018). Pioneering methods mainly rely on alkyl molecules as soft ligands to form interdigitated structures between adjacent nanoparticles (Whetten et al., 1996; Korgel et al., 1998; Mueggenburg et al., 2007; Liao et al., 2011). Jaeger et al. reported the fabrication of monolayer superlattice sheets with close-packed dodecanethiol-capped gold nanospheres (Mueggenburg et al., 2007). It demonstrated that the quality of the superlattice can be determined by the evaporation kinetics and attractive interaction between the particles and interface. Short molecular ligands also provided intrinsic tensile strength by ligand-ligand interactions (e.g., physical interdigitation, electrostatic interaction and hydrogen bonding), leading to mechanically robust 2D superlattices with Young's modulus ranging from 3~39 GPa (Lin et al., 2003). C.B. Murray and D. V. Talapin et al. developed a general method to fabricate binary nanoparticle superlattices using air-liquid interfacial assembly (Talapin et al., 2009; Dong et al., 2010). Through the regulation of nanoparticle stoichiometry/shapes/compositons (Ye et al., 2013a), various 3D nanoparticle superlattices can be generated with a controlled lattice structure (Boles et al., 2016). Nevertheless, the small molecule ligand-based method is only restricted to small or spherical building blocks, which limited its spatial control over interparticle electromagnetic coupling over a broad range. In the last two decades, DNA-mediated assembly has emerged as an advance technique due to its unique Watson-Crick base-pairing ability and synthetically designable structure (Jones et al., 2015), allowing for DNA-programmed, DNA-origami-templated or “dry ligand-based” assembly approaches in superlattice fabrication (Chen and Cheng, 2012). C. A. Mirkin and O. Gang et al. reported the fabrication of 3D nanoparticle supracrystals from DNA-capped nanocrystals (Nykypanchuk et al., 2008; Park et al., 2008). Both methods have demonstrated the programmable crystalline structure through the design of a DNA linker and precise temperature control. Furthermore, Mirkin's group established basic materials design rules for regulating lattice structures (Macfarlane et al., 2011) and dictating photonic properties by either spacing control or crystal habit (Ross et al., 2015), enabling more than 30 exotic lattice structures that are difficult to obtain using conventional strategies (Lin et al., 2018). However, a DNA-programmable approach requires complex procedures with strict control of ionic concentration or hybridization temperature. Also, 2D monolayer superlattice that is stable in dehydrated states can only achieved by using single strand DNA as the dry ligand and is limited to nanosphere building blocks (Cheng et al., 2009). Compared with small molecule and DNA based strategies, a recently developed polymer-ligand-based strategy constitutes a low-cost and scalable avenue to fabricate a 2D monolayer superlattice. P. D. Yang et al. fabricated densely-packed 2D superlattices using poly(vinyl pyrrolidone)-capped Ag nanoparticles, and demonstrated the tunable plasmonic properties by controlling surface pressure during assembly (Tao et al., 2007, 2008). W. L. Cheng et al. established a polystyrene-based technique and achieved free-standing superlattices by multiple constituent nanoparticles with complex shapes, which enables both structure regulation and property control (Chen et al., 2011; Si et al., 2014). By using a polymer-based strategy, new concepts and 2D superlattices have been reported in this burgeoning research area, such as plasmene-based origami (Si et al., 2014, 2016) and Janus superlattice (Shi et al., 2019b). With the integration of top-down nanofabrication approaches, the polymer-ligand-based strategy suggests a facile yet efficient pathway toward the fabrication of practical device construction (Shi and Cheng, 2020).

Due to the localized surface plasmons within nanocrystals and largely enhanced electromagnetic field in the interstitial spaces (Yan et al., 2009; Yap et al., 2012), such a plasmonic superlattice membrane will trigger potential applications such as SERS through the combination of the unique advantages from various noble metals (Alvarez-Puebla et al., 2011). Various techniques have been developed for fabrication of SERS substrates, thus achieving the detection of multiple-analytes (Cecchini et al., 2013), *in vivo* targets (Song et al., 2012), and even down to single-molecular substrates (Nie and Emory, 1997). Due to its high sensitivity, selectivity, and stability (Sharma et al., 2012), SERS has performed as a promising platform in biomedical and clinical applications, such as through the detection of biomarkers (Singhal and Kalkan, 2009; Hamon and Liz-Marzán, 2018), DNA/RNA (Barhoumi et al., 2008), virus and bacteria (Bodelón et al., 2016), hormones (Cho et al., 2018) etc. Such a SERS based bioassay is mainly achieved by two strategies—Raman dye-induced indirect method (Han et al., 2009a) and label-free direct ditection (Han et al., 2012; Xu et al., 2014; Cho et al., 2018). Compared with the dye-labeled method, label-free sensing can identify targeted molecules directly, by their distinctive vibrational and rotational signatures without complicated modification, which is paving a promising avenue for real-time detection. However, the sensitivity and signal uniformity of label-free detection still requires improvement due to the relatively low concentrations of biomolecules in the sample. It is therefore highly desirable to optimize the electromagnetic field enhancement by adjusting the nanostructures, minimizing the sophisticated process involved in nanofabrication, and maximizing uniformity applicable for reproducible and robust signal (Osberg et al., 2012).

Despite the exciting advances in producing plasmonic structures achieved thus far, it is clear that undeveloped issues remain ahead. It has been remarkably difficult to manipulate at will for the large-scale, ordered assembly of plasmonic nanoparticles due to complex interparticle forces. Structure defects severely limit their tunable plasmonic property, thus restricting their practical applications. The fabrication of novel plasmonic membranes with controllable properties and multiscale topology is trending and remain critical challenges for the field of self-assembled metamaterials. Herein we report on the hierarchical fabrication of large-area, free-standing plasmonic superlattice membranes using aspect-ratio programmable ultralong Au@Ag core-shell nanobricks (NBs) as building blocks. Such bimetallic NBs are synthesized using long Au nanorods as the core, with tunable SPR band ranges from the visible to near-infrared region, followed by a uniform silver coating. Three distinctive plasmonic superlattice membranes were constructed *via* the general ligand-based method in conjunction with drying-mediated self-assembly. Detailed morphology characterization reveals the 2D monolayered structure with macroscopic lateral dimension as well as nanoscopic ordering (about 5-mm-wide and 60-nm-thick, corresponding to an aspect ratio of 80,000). We also demonstrate that the fabricated membrane exhibits a nearly uniform SERS signal across their surface and in turn indicates homogenous hot-spot distribution, which makes them perfect SERS substrates for label-free detection of proteins. By using 4-ATP as a trace chemical and BSA as a model protein, we evaluated its high-sensitive SERS performance and proved the label-free SERS detection of BSA with a detection limit of 15 nM. The SERS enhancement factor (EF) is calculated to be 4.3 × 10^5^, with a relative standard deviation of about 3.4%, indicating a high sensitivity while maintaining excellent signal uniformity. We believe our methodology may serve as a promising general approach with great modularity and versatility toward fabricating 2D plasmonic membrane from other nanomaterials and opens vast possibilities for a myriad of applications in construction of meta-devices and sensors for biomedical application.

## Materials and Methods

### Chemicals and Materials

Gold (III) chloride trihydrate (HAuCl_4_·3H_2_O, ≥99.9%), hexadecyltrimethylammonium bromide (CTAB), cetyltrimethylammonium chloride solution (CTAC, 25 *wt*% in H_2_O), and L-ascorbic acid (AA, ≥99%) were purchased from Sigma Aldrich. Sodium Oleate (NaOL, 98%), sodium borohydride (NaBH_4_, ≥99%), silver nitrate (AgNO_3_, 99.99%), Hydrochloric Acid (HCl, 37 *wt*% in H_2_O), Tetrahydrofuran (THF, 99%) and Chloroform (99%) were obtained from MACKLIN. Thiol-functionalized polystyrene (*M*_*n*_ = 50,000 g mol^−1^, *M*_*w*_/*M*_*n*_= 1.06) was obtained from Polymer Source Inc. Bovine serum albumin (BSA, Fraction V) was obtained from Solarbio. Quantifoil holey carbon films (400 mesh with 37 μm × 37 μm square holes) and Gilder Square Grids (2,000 mesh with 7.5 × 5 μm^2^ and 300 mesh with 54 × 54 μm^2^ square holes) were purchased from Zhongjingkeyi Technology Co., Ltd.

All chemicals were used as-received unless otherwise indicated. Demineralized water was used in all aqueous solutions, which were further purified with a Milli-Q system (Millipore). All glassware used in the following procedures were cleaned in a bath of freshly prepared aqua regia and were rinsed thoroughly in H_2_O prior to use.

### Synthesis of Aspect-Ratio Controllable Gold Nanorods (AuNRs)

The synthesis of AuNRs was prepared by adopting previously reported approaches with slight modification (Ye et al., 2013b). First, the seed solution of gold nanospheres was synthesized by mixing 5.0 ml 0.2 M CTAB with 5.0 ml 0.5 mM HAuCl_4_, followed by quickly adding 0.6 ml 0.01 M ice-cold NaBH_4_ under vigorous stirring. The seed solution showed a brownish color. To prepare the growth solution, 0.7 g CTAB and 0.1234 g NaOL were dissolved in 50 ml hot water. Then 2.4 ml of 4 mM AgNO_3_ solution was added to the above solution and left undisturbed for 10 min. Then 1 ml of 25 mM HAuCl_4_ solution was added, the solution for stirring for more than 45 min until the solution turned colorless. Then a small amount of HCl (37 wt%) was added to adjust the pH of the growth solution and stirred for 15 min. By increasing the amount of HCl from 0.5 to 0.8 ml, the aspect ratio of AuNRs can be finely controlled. After adding 125 μl of 64 mM ascorbic acid solution, the growth solution was violently stirred for 30 s, followed by adding 80 μl of seed solution, then the mixture was left steady at 30°C for more than 12 h. The final solution was centrifuged at 7,000 rpm for 25 min and re-dispersed in 50 ml water.

### Synthesis of Aspect-Ratio Controllable Au@Ag Nanobricks (NBs)

The as-prepared gold nanorods solution was centrifuged at 7,000 rpm for 20 min and re-dispersed in 10 ml 80 mM CTAC solution. This ligand-exchange process was repeated twice to ensure complete replacement of CTAB ligands by CTAC capping ligands. Then a silver precursor solution was added into the as-prepared CTAC-capped AuNRs to enable the seed-mediated growth process. Typically, a mixture of 880 μl AgNO_3_ (0.01 M) and 440 μl ascorbic acid (0.1 M) was mixed with 10 ml CTAC-capped AuNRs. The resultant solution was kept incubated at 65°C for 3 h under stirring conditions. After that, the solution was centrifuged at 7,000 rpm for 20 min and re-dispersed in deionized water for further use.

### Ligand Exchange From CTAC- To Polystyrene-Capped Au@Ag NBs

As-prepared CTAC-capped Au@Ag NBs (10 ml) was centrifuged and concentrated into 200 μl, followed by adding 3 ml of thiolated polystyrene-THF solution (2 mg ml^−1^) under vigorous stirring. After aging overnight, the supernatant was discarded, and samples were purified by repeated centrifugation–precipitation cycles with chloroform. Finally, the PS-capped Au@Ag NBs were re-dispersed in chloroform for membrane fabrication.

### Fabrication of Plasmonic Superlattice Membranes

A droplet-based interfacial assembly technique was used for the preparation of large-area superlattice membranes on substrates such as bare TEM copper grid (2,000 mesh) or silicon wafer. Typically, one droplet (~3 μl) of as-prepared PS-capped NBs was carefully spread onto the surface of convex-shape water droplet sitting on the substrate at ambient temperature. Immediately after the quick spreading over the water surface, the substrate was then covered to prevent fluid flows and solvent fluctuations. After 5 min of equilibration, a silver-colored reflective membrane covered on the water subphase, indicating the complete solidification. After the evaporation of water, the superlattice membrane covered almost the whole substrate.

### Characterization

Morphology characterization was carried out using HITACHI HT7700 TEM and FEI Quanta 250 FEG SEM. The height profile was obtained using Veeco Dimension Icon AFM in tapping mode. The optical extinction spectra of nanoparticles were measured by Shimadzu SolidSpec-3700 UV-Vis spectrophotometer. The surface of the membrane was treated for 5 min by plasma cleaner (PDC-MG) to remove the top ligands before SERS detection. The data analysis of the TEM images was conducted by ImageJ 1.50b.

### Label-Free Detection of BSA

The superlattice membranes were immersed in BSA aqueous solution with different concentrations for 12 h. Then, the superlattice membranes were rinsed with water to remove free BSA on the surface after the complete evaporation of water, the superlattice membranes were ready for SERS measurements.

### SERS Measurements

SERS spectra were acquired by using Thermo Scientific DXR 2Xi Raman Imaging Microscope with a diode laser at 633 nm. A laser power of 2 mW was used for all measurements. The exposure time of the laser on the sample was 10 s.

## Results and Discussion

**Figure 1:**
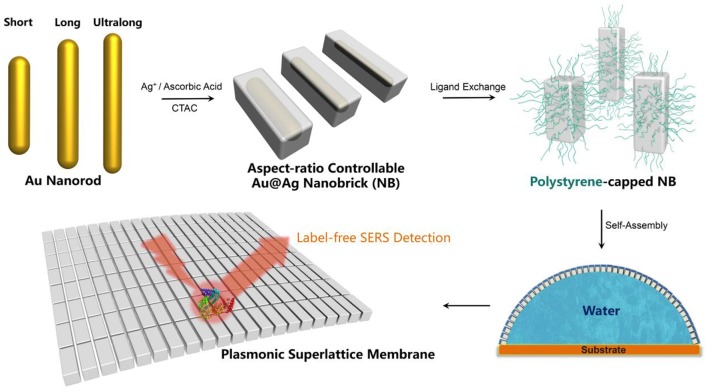
Schematic illustration for the fabrication of plasmonic superlattice membranes by aspect-ratio controllable nanobricks (NBs).

**Figure 2:**
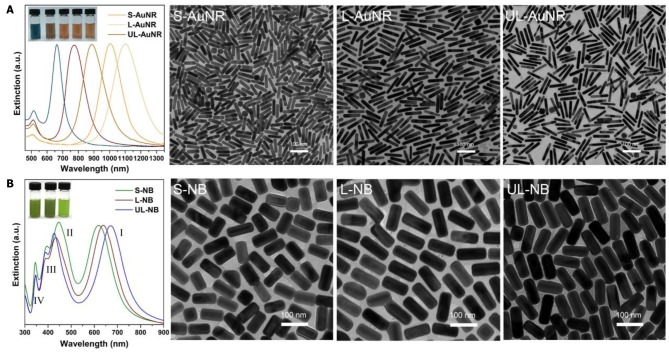
High-quality synthesis of aspect-ratio controllable nanoparticles. **(A)** Extinction spectra of five synthesized Au nanorods with longitudinal SPR bands ranging from 663 to 1,103 nm. Among them, three AuNRs were chosen as templates to synthesize nanobricks. TEM images indicate the well-defined morphology of Short-, Long-, and Ultralong-AuNRs. **(B)** Extinction spectra and corresponding TEM images of Short-, Long-, and Ultralong-Au@Ag Nanobricks. The inset shows the photograph of AuNRs and NBs solution with distinct colors.

It is non-trivial to fabricate defect-free superlattice membranes without the synthesis of monodispersed plasmonic building blocks. Existing methods emphasize the sophisticated technique under optimized conditions but ignore the rational design of the essential constituent nanocrystals, and therefore hardly leads to a large-area membrane with nanoscale thickness and macroscopic lateral dimensions. The starting point of fabricating the plasmonic superlattice membrane was the synthesis of high-quality ensembles of ultralong nanobricks through the modification of a recently developed method (Okuno et al., 2010), followed by the replacement of ligands attached to the nanoparticles during the synthesis (Figure 1). We first synthesized highly monodisperse nanoparticle cores in the form of short-, long- and ultralong-gold nanorods (AuNRs) with tunable SPR bands ranging from the visible to near-infrared region. Among them, three AuNRs with different aspect ratios were chosen as template cores, corresponding to characteristic longitudinal SPR bands at 888, 1,006, and 1,103 nm (Figure 2A). The three AuNRs were then coated by uniform layers of silver by controlling the stoichiometric amount of Ag^+^ precursor and nucleation cores (Table S1 and Figure S1). The so-prepared Au@Ag core-shell nanobricks (NBs) exhibited well-pronounced longitudinal/transverse dipolar (peak I and II), and octopolar (peak III and IV) localized surface plasmon modes with spectral positions strongly dependent on the AuNRs core and silver shell (Figure 2B). With the increase of the aspect ratio, the three NBs were nominated as short (S-NBs), long (L-NBs), and ultralong (UL-NBs) NBs. The transition of plasmonic properties can be directly visualized from the varying colors of different NBs, which was further monitored by UV-Vis spectroscopy. It indicates that the SPR band peak I maintains the blue-shift with the increasing aspect ratio. Since the concentration of AgNO_3_ is constant during the synthesis, the tunable optical response is mainly induced by the longitudinal dipolar plasmon mode of AuNR core and end-to-end silver shell. Based on the TEM characterization and detailed analysis (Figure 2), the NBs exhibited uniform size distribution and customizable morphology (Table S1 and Figure S2). Subsequently, ligand exchange and phase-transfer were achieved by preparing polystyrene (PS)-capped NBs using a two-step grafting procedure. Thiolated-PS ligands (*Mn* = 50,000 g mol^−1^) successfully replaced the weaker-binding cetyltrimethylammonium chloride (CTAC) *via* a robust covalent linkage between the PS and the silver shell. The hydrophobic property of PS ligands effectively stabilizes the NBs and prevents them from aggregating in chloroform (Corbierre et al., 2004). Such a well-dispersed form is a prerequisite for the following self-assembly process.

**Figure 3:**
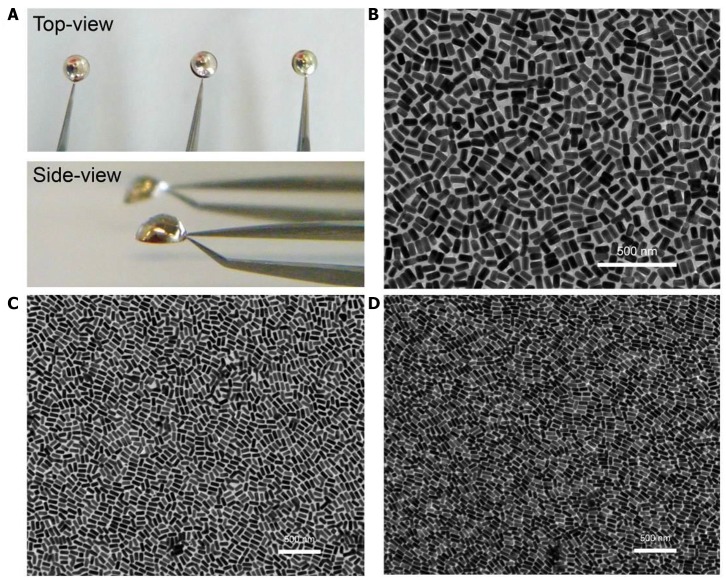
Morphological characterization of plasmonic superlattice membranes. **(A)** Photograph of membrane self-assembly at the air/water interface. **(B–D)** TEM images of S-NB, L-NB, and UL-NB plasmonic membrane, respectively.

The plasmonic superlattice membrane was fabricated using a ligand-based strategy and drying-mediated air-liquid interfacial self-assembly. After repeated centrifuging and concentrating, one droplet of the as prepared PS-NBs solution was carefully spread onto the surface of a convex-shaped water droplet sitting on the substrate at ambient temperature (Figure 1). During the rapid evaporation of chloroform, the strong surface tension confined the monolayered membrane constructed by the self-assembling PS-NBs to the air/liquid interface. After 5 min of equilibration, a silver-colored reflective membrane covered the water subphase, indicating the complete solidification and localization of NBs (Figure 3A). Due to the subsequent slow evaporation of water, the plasmonic membranes fused and covered the substrate surface. During the self-assembly process, the soft and long PS ligands provide sufficient steric hindrance forces to balance the strong core-to-core van der Waals attractions between NBs along with the lateral capillary force applied by the surface tension of the evaporating chloroform, which facilitate the ordered packing for nanoassemblies (Yockell-Lelièvre et al., 2007; Bishop et al., 2009). By applying de Gennes's polymer brush theory (de Gennes, 1980) and entropic spring model (Cheng et al., 2010) during the soft-crystallization process, we evaluated the softness (χ) and deformation parameter (λ) of the PS ligand, which reaches a deformation degree of 0.7 and demonstrates the strong entanglement and compression in the lateral direction during the assembly process (Table S2).

**Figure 4:**
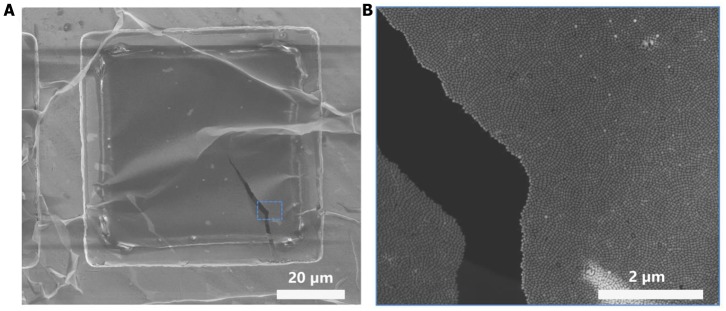
SEM characterization of plasmonic superlattice membrane. **(A)** SEM image at low magnification shows the plasmonic membrane covering the square-shaped holey copper grid. The membrane exhibits crumpled and ridge-like morphology, demonstrating high flexibility, and robustness. **(B)** Higher-magnification SEM image of the ruptured rectangular region in **(A)**.

**Figure 5:**
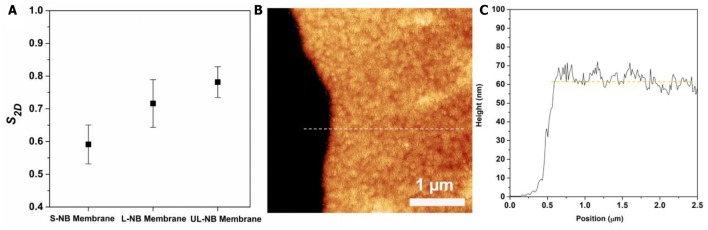
**(A)** The calculated 2D orientational order parameter (*S*_2*D*_) of three plasmonic membranes. **(B)** AFM height image of the plasmonic membrane on silicon wafer. **(C)** AFM cross-sectional height plot corresponding to the dashed line in **(B)**.

The plasmonic superlattice membranes consisted of two-dimensional close-packed Au@Ag NBs arrays that uniformly continuous extending to centimeter-scale. By using a holey copper grid (300 mesh with 54 × 54 μm^2^ holes) as substrate during fabrication, thorough morphological and topological characterization was carried out using transmission electron microscopy (TEM), a scanning electron microscope (SEM) and an atomic force microscope (AFM). TEM images in Figure 3 show that the membranes are continuous and uniform at the nanoscale, indicating horizontally aligned NBs with well-separated interparticle spaces and a unique monolayered structure. The edge-to-edge interparticle distances have been statistically analyzed from TEM images (Figure S3). The interparticle spacing is 8.3, 8.9, and 8.2 nm for S-, L-, and UL-NB membranes, which indicates uniform distributions of interparticle distances due to the compression of the PS ligands. Furthermore, SEM characterization exhibits that the giant plasmonic membrane covered the holey copper grid with a rippled and ridge-like appearance, suggesting robustness and flexibility within large-area domains (Figure 4A). SEM image at higher magnification shows the fractured domain of membrane, which indicates the monolayered structure with closely packed NBs (Figure 4B). Unlike spherical building blocks, the anisotropic NBs are horizontally aligned in a more complex way. To quantify the degree of ordering for the NBs plasmonic membrane, the 2D orientational order parameter (*S*_2*D*_) was calculated using Hore's method (Hore and Composto, 2010):

$$\begin{array}{l} S_{2D}=\frac{1}{N_{NBs}}\overset{N_{NBs}}{\underset{i=1}{\sum }}\text{ cos }2\theta_{i} \end{array}$$

where θ_*i*_ is the angle between the *i*th NB and the average orientation of NBs in a selected region of radius *r* around it, and *N*_*NBs*_ is the total number of nanoparticles within the analyzing region (Figure S4). The *S*_2*D*_values for three membranes were calculated from TEM images, which indicates an increasing trend from S-NB to UL-NB membrane (Figure 5A). Such increasing ordering can be explained by more side-by-side packing for long NBs with large aspect-ratios (Ng et al., 2011). Further AFM characterization and line scanning shows an average thickness of 62 ± 3 nm, which is comparable to the width of constituent PS-capped NBs (Figure 5). The AFM height image indicates the monolayered 2D topography of the plasmonic membrane, which is evidently in agreement with the TEM and SEM characterization. Remarkably, the plasmonic membrane shows a considerable surface area that reaches a lateral size of about 5 mm, while the thickness is close to single nanoparticle width, thus featuring a substantial width-to-thickness aspect ratio of about 80,000.

**Figure 6:**
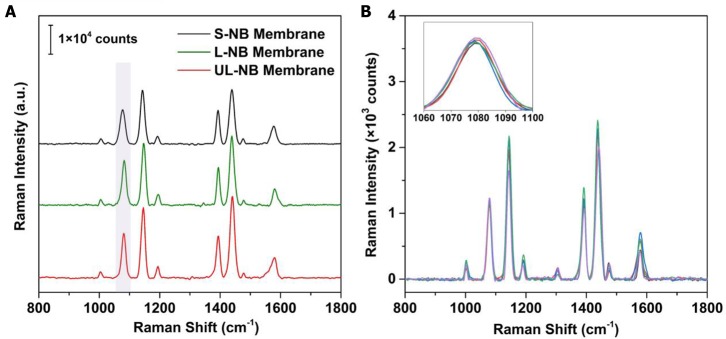
SERS performance of plasmonic superlattice membranes. **(A)** Evaluation of SERS efficiency by comparing 4-ATP spectra on three membranes under the same 4-ATP concentration of 1 μM. **(B)** SERS spectra obtained from 5 different spots on the surfaces of UL-NB membrane after exposure to trace 4-ATP solution (100 nM). The overlapped spectra indicate high homogeneity of SERS detection.

Plasmonic superlattice membranes exhibit tailorable SERS efficiency due to the alteration of constituent nanoparticles and structure-dependent conformation. To evaluate the SERS performance, we used 4-aminothiophenol (4-ATP) as a model SERS probe owing to its high affinity to silver and its large Raman cross-section. Three plasmonic membranes were exposed to trace amounts of 4-ATP under the same concentration and then irradiated by laser under an excitation wavelength of 633 nm. By comparing the Raman intensities of the characteristic peak at 1,078 cm^−1^, it evidently indicated that the SERS efficiency was gradually increasing from the S-NB to the UL-NB membrane (Figure 6A). Since the SERS enhancement factor (EF) is proportional to the fourth power of the electromagnetic confinement strength, EF ∞ (|**E**|/|**E**_**0**_|)^4^, it proved that the UL-NB membranes possess the most robust near-field confinement due to the ultralong morphology of NBs and enhanced plasmonic coupling.

A unique feature of the plasmonic superlattice membranes is their high structural homogeneity and the resulting uniformity of the SERS signal across the surface. This feature was demonstrated by recording and superimposing SERS spectra of 4-ATP from various spots (of about 0.8-μm^2^ each) on the surface of the membrane (Figure 6B). The overlap of the Raman spectra indicates the outstanding uniformity of the SERS signal. By extracting the intensities for the 1,078 cm^−1^ peak, the maximum EFs can reach 4.3 × 10^5^, with a relative standard deviation of 3.4%. This variance is well-below those reported SERS substrates such as patterned Ag-arrays (5%) (Yakun et al., 2018) or nanopillar chip (4.5%) (Zhao et al., 2015).

**Figure 7:**
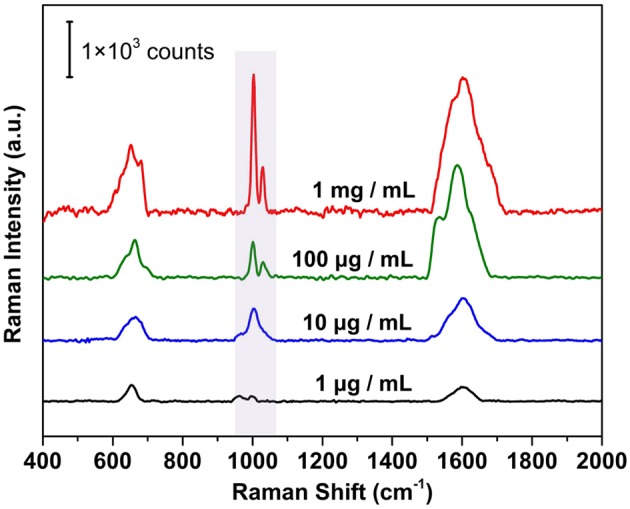
Label-free SERS detection of BSA under different concentrations ranges from 1 mg/mL to 1 μg/ml.

The combination of high sensitivity and uniformity from the plasmonic membrane suggests that they are practical and versatile SERS substrates for label-free protein detection. The SERS based label-free strategy shows unique advantages for facile yet efficient identification of the inherent vibrational properties of the biomolecules. For proteins with chromophores (Han et al., 2009b) (e.g., cytochrome c, hemoglobin, and myoglobin), it is relatively straightforward to obtain the signal related to the conformation and orientation of the protein. However, for most proteins without chromophores, it is challenging to obtain the weak signals induced by amino acid residues and amide backbones, which is rarely achieved by conventional SERS substrates. We demonstrated this possibility by measuring the SERS spectrum of bovine serum albumin (BSA) as a model protein, which contains a negatively charged amino acid domain (glutamic acid or aspartic acid) and positively charged domain (lysine or histidine). After removing the organic ligand and contaminants by UV-ozone treatment (Figure S5), the pristine membrane was exposed to BSA aqueous solution with different concentrations. Attributing to the electrostatic adsorption and Ag-S covalent bond induced by the cysteine residue in BSA, the BSA deposited on the membrane surface and was ready for SERS detection. One can see from Figure 7 that the SERS spectra show a trend of decreasing in Raman intensity with decreasing loading concentrations. In particular, a decrease in peak intensity was observed when the BSA concentration was reduced from 1 mg/ml to 1 μg/ml for the characteristic 1,000 cm^−1^ band identified from bulk BSA powders (Figure S6). The characteristic peak mainly originated from the vibration of tryptophan and phenylalanine in the BSA structure (Xu et al., 2014), which is assigned to the breathing mode (ν_12_) of phenyl ring (Zhang et al., 2014). When the BSA concentration was reduced to 1 μg/ml, the intensity of the characteristic peak was already very weak, indicating a detection limit of 15 nM for our plasmonic superlattice membrane.

## Conclusions

In summary, this paper presents a simple yet efficient strategy to fabricate plasmonic superlattice membranes of millimeter-sized surfaces and nanoscale thicknesses, using aspect-ratio controllable ultralong nanobricks as building blocks. We demonstrated that superlattice membranes assembled by short/long/ultralong nanobricks could be fabricated in the form of free-standing, ordered, monolayer topology, which is flexible and robust enough to be readily used as high-performance SERS substrates. Rational control over the constituent nanocrystals allows for customizable SERS efficiency. Being a 2D quasi-periodic array of interconnected bimetallic nanoparticles, the plasmonic membranes possess high structural homogeneity and exhibits near-field confined hotspots uniformly fashioned over the whole substrate. Owing to these features, it enables ultrasensitive SERS-based detection of trace chemicals and label-free direct detection of BSA, with the detection limit reaching as low as 15 nM. Our strategy may represents a significant step toward the exploitation of plasmonic membranes for a plethora of technical applications such as next-generation of nanophotonics devices and point-of-care biosensors, since plasmonic superlattice fabrication and the ability to nanoengineer its SERS performance as required in practice, by varying the sizes, shapes, and compositions of the constituting nanoparticles is easy.

## Data Availability Statement

All datasets generated for this study are included in the article/Supplementary Material.

## Author Contributions

YC and NG conceived and planned the experiments, which were carried out by YC, HL, HY, QZ, and GY. YC, NG, HL, and HY analyzed the experimental data. YC, HL, and HY co-wrote the paper. YC, HL, HY, and NG discussed the results and comprehensively revised the manuscript. All authors approved its submission.

## Conflict of Interest

The authors declare that the research was conducted in the absence of any commercial or financial relationships that could be construed as a potential conflict of interest.
