# Supplementary material for: Hierarchical Fabrication of Plasmonic Superlattice Membrane by Aspect-Ratio Controllable Nanobricks for Label-Free Protein Detection
Source: Front Chem. 2020 Apr 28;8:307. doi: 10.3389/fchem.2020.00307 (PMC7198893; doi:10.3389/fchem.2020.00307)
Supplement: Supplementary file 1 [file Data_Sheet_1.pdf]

# ***Supplementary Material***

## Table of Contents

Section 1. Controllable Synthesis of High-quality Nanoparticles ........................... 3

Section 2. Analytical Characterization of Plasmonic Membranes........................... 6

*2.1 Measurement of interparticle distance* ........................................................ 6

*2.2 Calculations on softness and deformation parameter of PS ligand* ............... 6

*2.3 Calculations on ordering parameter of plasmonic membrane*....................... 8

Section 3. SERS Characterization and Evaluation.................................................. 9

*3.1 Raman spectra of relevant characterization*................................................. 9

*3.2 Calculation of SERS enhancement factor*....................................................11

4 References ..........................................................................................................13

1 **Section 1. Controllable Synthesis of High-quality Nanoparticles**

**Table S1** Detailed dimensions of Au Nanorods and Au@Ag Nanobricks

| Entry | | Length [b] ($l$, nm) | Diameter [b] ($d$, nm) | Aspect ratio |
|---|---|---|---|---|
| Au nanorod (core) | **S-AuNR** | 72.4 ± 5.8 | 16.5 ± 1.7 | 4.5 ± 0.6 |
| | **L-AuNR** | 97.8 ± 8.3 | 16.6 ± 1.0 | 5.9 ± 0.6 |
| | **UL-AuNR** | 98.6 ± 7.6 | 14.9 ± 1.2 | 6.6 ± 0.8 |
| Entry [a] | | Length [b] ($l$, nm) | Width [b] ($w$, nm) | Aspect ratio |
| **S-NB** | | 85.0 ± 7.2 | 41.4 ± 3.1 | 2.1 ± 0.3 |
| **L-NB** | | 105.7 ± 6.1 | 42.9 ± 1.7 | 2.5 ± 0.2 |
| **UL-NB** | | 103.4 ± 8.4 | 40.4 ± 2.2 | 2.6 ± 0.3 |

*a: The ligands for NBs were thiol-functionalized polystyrene (Mn=50,000, $M_w/M_n$ = 1.06); b: Obtained from TEM images of AuNRs and Au@Ag NBs, and statistical analysis through the free software ImageJ, as shown in following figures.*

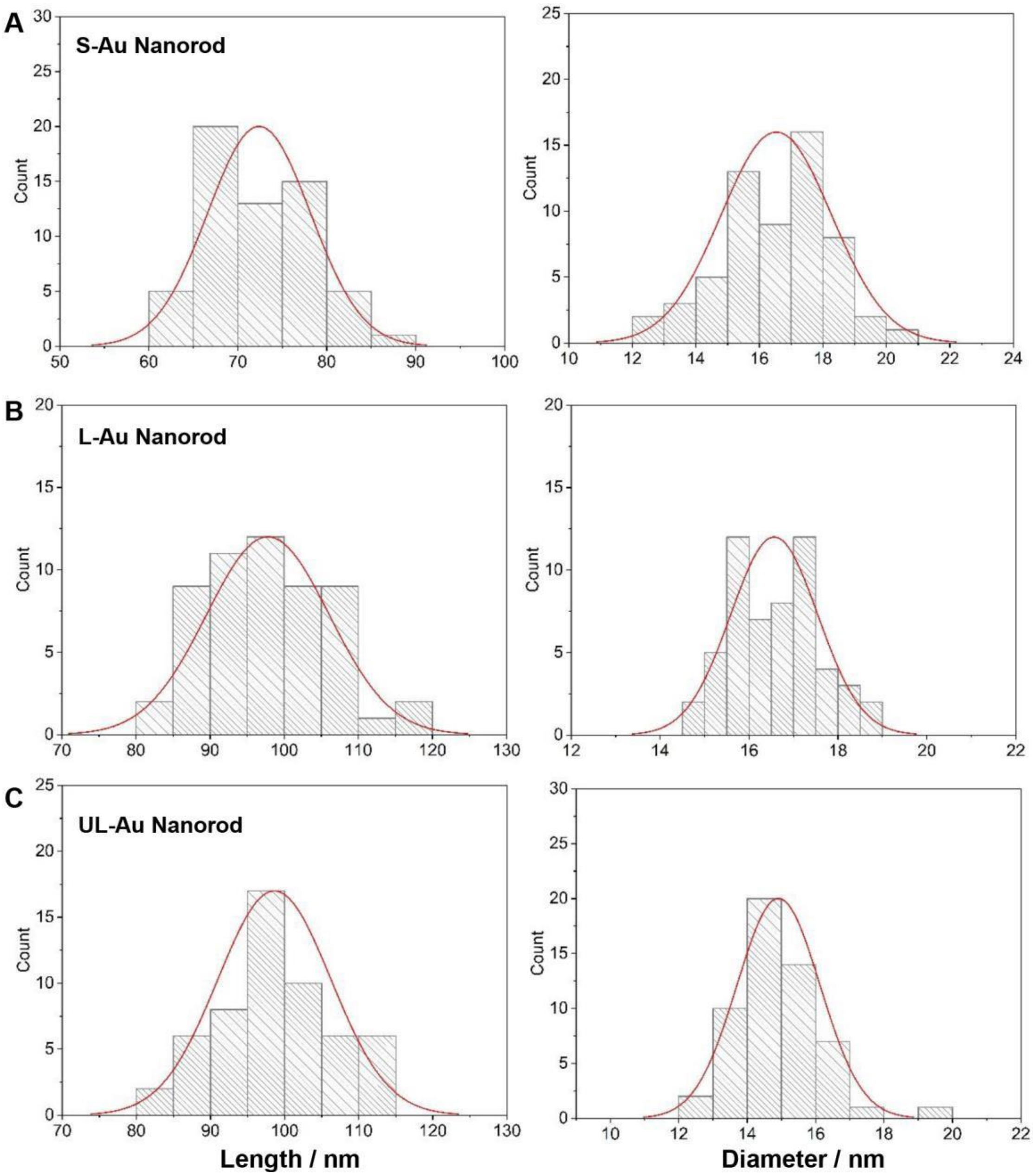


**Figure S1**. Statistical analysis showing distribution of length and diameter of Short-, Long-, Ultralong-AuNRs measured from TEM images. Red curves represent the Gaussian fits to the distributions.

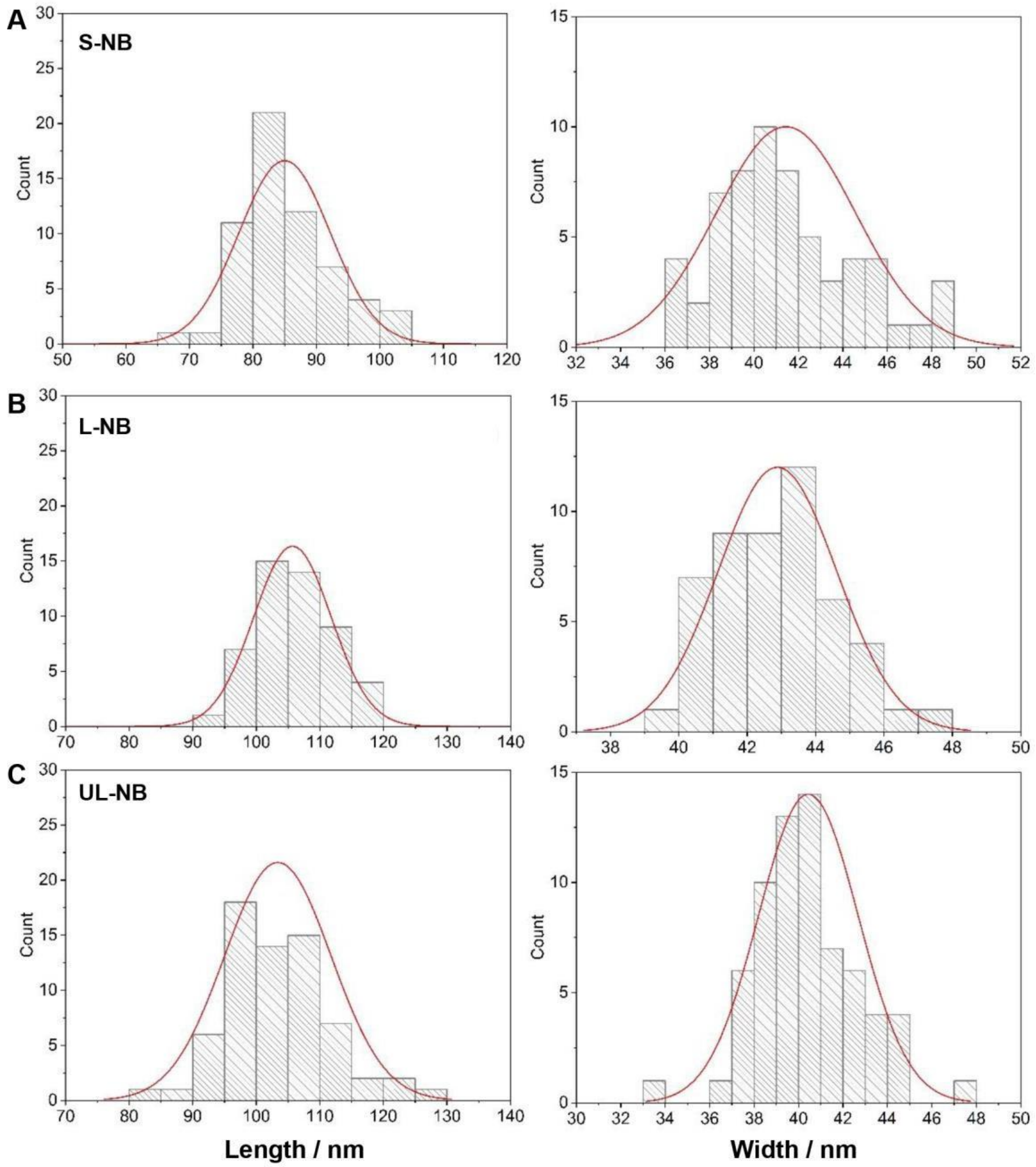


**Figure S2**. Statistical analysis showing distribution of length and width of Short-, Long-, Ultralong-Au@Ag NBs measured from TEM images. Red curves represent the Gaussian fits to the distributions.

## 2 Section 2. Analytical Characterization of Plasmonic Membranes

### *2.1 Measurement of interparticle distance*

Based on the TEM characterization of the three plasmonic membranes, we measured the interparticle spacing of NBs (*DNN*) and fitted by Gaussian approach.

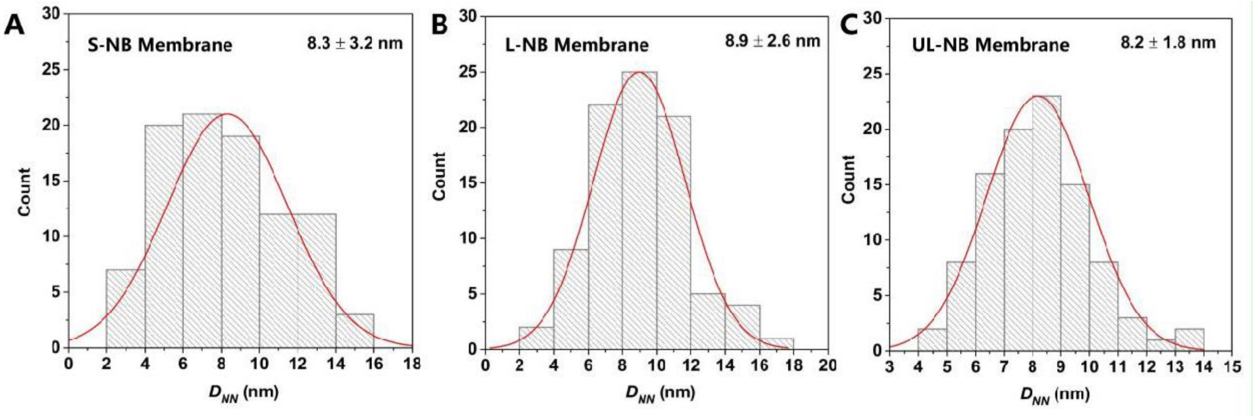


**Figure S3**. Statistical analysis of interparticle distance of NBs for three plasmonic membranes measured from TEM images. Red curves represent the Gaussian fits to the distributions.

### *2.2 Calculations on softness and deformation parameter of PS ligand*

Before the self-assembly, the polystyrene-capped NBs were well-dispersed in chloroform. The densely capped PS ligands adopt a brush conformation on the surface of NBs. By using de Gennes's model[1], the hight of PS ligand ($h_0$) at solvated state can be pridicted by the following scaling equation[2],

$$h_0 = Na(a/d)^{2/3} \quad (1)$$

where $N$ is the degree of polymerization, $a$ is the average Kuhn length, $d$ is the chain footprint diameter. The calculated *h0* for the PS-capped NBs is equal to 72nm.

During the self-assembly process, the soft PS ligands successfully balanced core-core Van der Waals attraction and lateral capillary forces by experiencing great compression in the lateral directions. By applying the entropic spring model that established for soft-ligand guided assembly process[3], the deformation of PS chains can be quantified. The softness of the PS-NBs can be evaluated by the dimensionless quantity $\chi$,

$$\chi=2h_0/w \tag{2}$$

where $h_0$ is the equilibrium height of the PS corona at hydrated state, and $w$ is the width of NB.

The deformation degree of PS could be further quantified by a dimensionless deformation parameter $\lambda$,

$$\lambda=\frac{2h_0-D_{NN}}{2h_0+w} \tag{3}$$

where $D_{NN}$ is the interparticle spacing of NBs in plasmonic membrane. The calculated data was summarized in Table S2.

**Table S2.** Analytical characteristics of PS ligand and plasmonic membranes

| Entry*a* | Edge-to-edge distance*b* (nm) | Softness*c* (χ) | Deformation parameter*c* (λ) |
|---|---|---|---|
| **S-NB Membrane** | 8.3 ± 3.2 | 3.5 | 0.7 |
| **L-NB Membrane** | 8.9 ± 2.6 | 3.4 | 0.7 |
| **UL-NB Membrane** | 8.2 ± 1.8 | 3.6 | 0.7 |

*a, The thiol-functionalized polystyrene with the same length was used as the ligand for all NBs (Mn=50,000, Mw/Mn=1.06, Degree of polymerization=480); b, Determined by TEM and statistical analysis in Section 2.1; c, the dimensionless quantity χ and deformation parameter λ were calculated by applying the entropic spring model in Section 2.2.*

### *2.3 Calculations on ordering parameter of plasmonic membrane*

Using the TEM image of UL-NB membrane as example, circular regions were randomly selected for analysis, as shown in **Figure S4**. For each region, *S2D* is calculated with r = 150, 300, ... , 1500 nm and plotted in **Figure S4B**. When the radii are above ~600 nm, the *S2D* reached a plateau of about 0.77. This value represented the degree of ordering of plasmonic membrane.

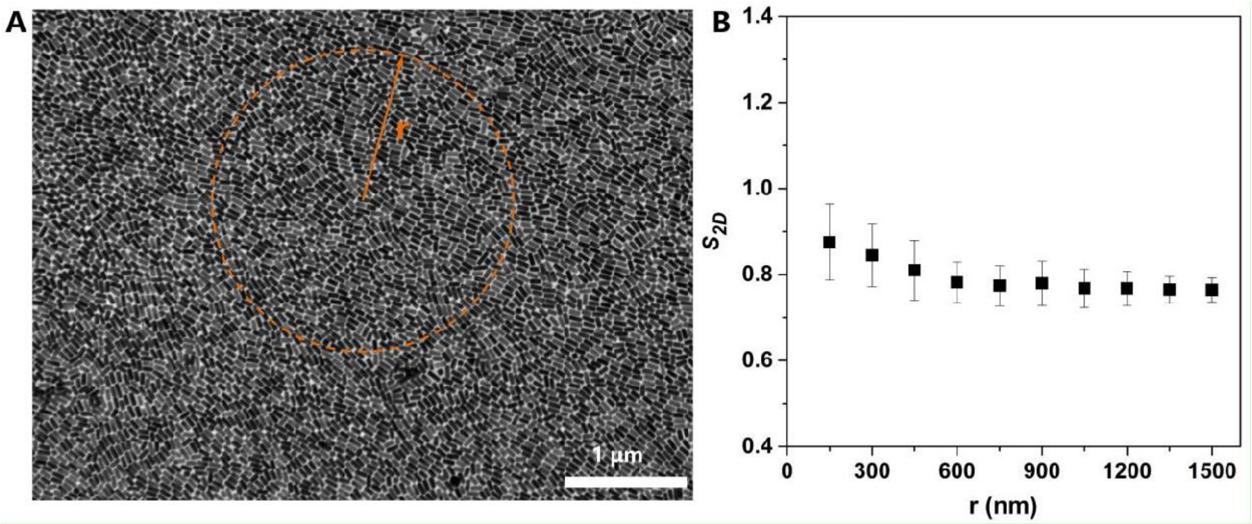


**Figure S4**. (A) TEM image of UL-NB plasmonic membrane. (B) 2D orientational-order parameter *S2D* plotted as a function of the radius (*r*) of the analyzed region.

# 3 Section 3. SERS Characterization and Evaluation

## *3.1 Raman spectra of relevant characterization*

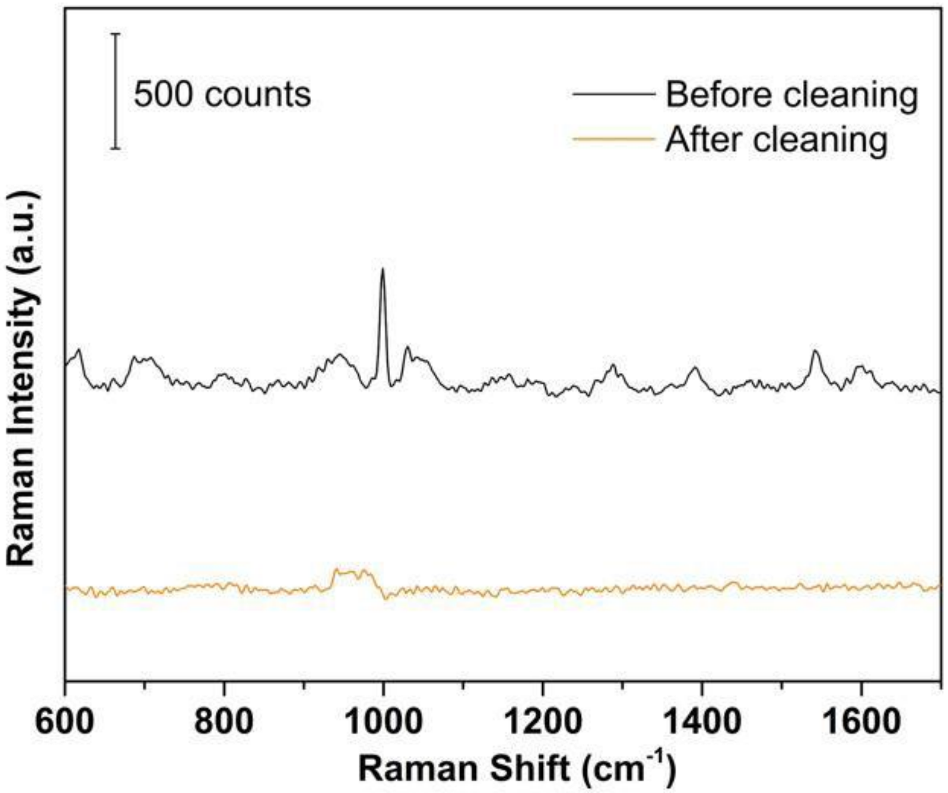


**Figure S5**. Raman spectra of superlattice membrane before and after plasma treating.

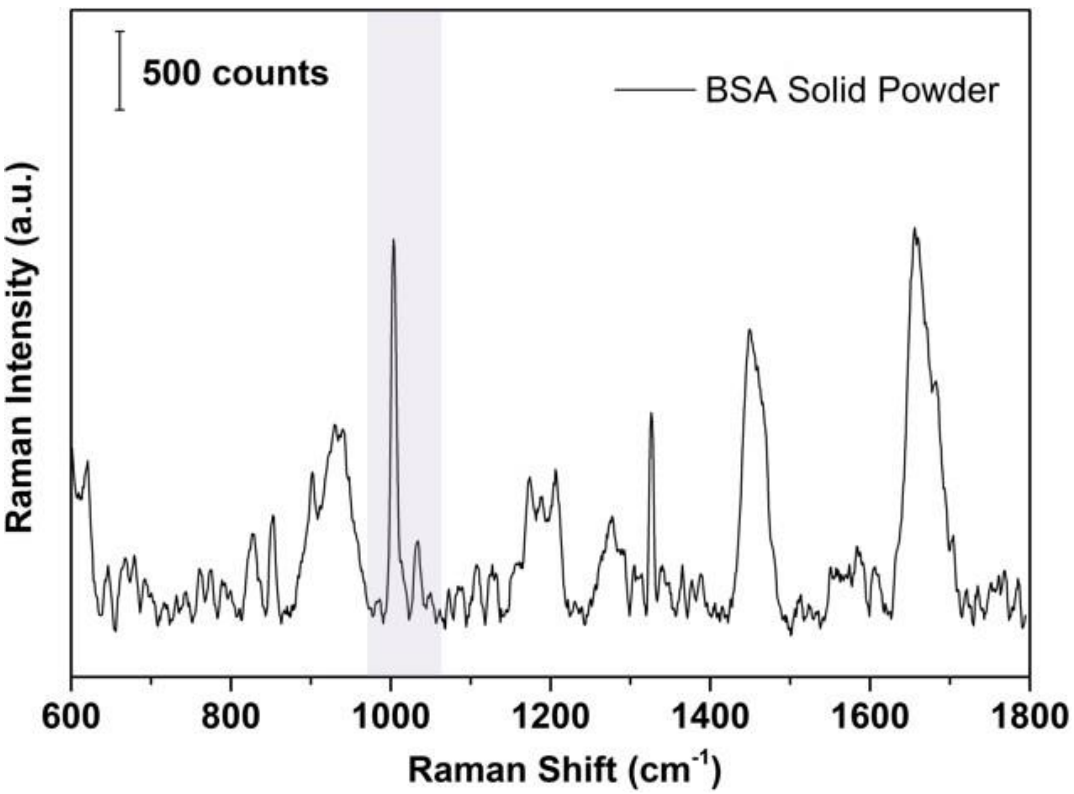


**Figure S6**. SERS spectra of BSA solid powder.

### 3.2 Calculation of SERS enhancement factor

4-ATP has characteristic vibration bands in the region between 800 and 1800 $cm^{-1}$. These peaks are mainly assigned to either the $a_1$ or $b_2$ vibrational modes[4] (Table S3). The energetic vibrational band at ~1078 $cm^{-1}$ was used to calculate SERS enhancement factor (EF) which was determined by the following equation (4):

$$EF = \frac{I_{SERS} / N_{SERS}}{I_{BULK} / N_{BULK}} \tag{4}$$

where $I_{SERS}$ and $I_{BULK}$ are the peak intensity at 1078 $cm^{-1}$ in the SERS spectrum of 4-ATP and bulk 4-ATP, respectively; $N_{SERS}$ is the number of adsorbed 4-ATP molecules on the substrate within the laser spot; $N_{BULK}$ is the number of 4-ATP molecules within the illumination volume of laser in a bulk sample. $N_{SERS}$ can be further calculated by:

$$N_{SERS} = N_d A_{laser} A_N / \sigma \tag{5}$$

where $N_d$ is the number density of particles per unit area. Based on the morphology characterization from TEM, the $N_d$ was calculated to be $1.8\times10^{-4}$, $1.4\times10^{-4}$ and $1.7\times10^{-4}$ particles/$nm^2$ for S-NB, L-NB and UL-NB membrane, respectively. $A_{laser}$ is the area of the focused laser spot, $A_N$ is the nanoparticle surface area determined by TEM measurements and σ is the footprint size of 4-ATP molecule (~ 0.20 $nm^2$)[5].

**Table S3** Raman spectral peak assignment for 4-ATP adsorbed on plasmonic membranes

| 4-ATP Raman ($cm^{-1}$) | Vibrational assignment [a] |
|---|---|
| 1078 | νCS, 7a (a1) |
| 1141 | δCH, 9b(b2) |
| 1392 | νCC+δCH, 3(b2) |
| 1438 | νCC+δCH, 19b(b2) |
| 1578 | νCC, 8a(a1) |

***a**: ν and δ denotes stretching and bending respectively.*

## 4 4 References

1. de Gennes, P.G. Conformations of Polymers Attached to an Interface. *Macromolecules* **13**, 1069-1075 (1980).

2. Yockell-Lelièvre, H., Desbiens, J. & Ritcey, A.M. Two -Dimensional Self-Organization of Polystyrene-Capped Gold Nanoparticles. *Langmuir* **23**, 2843-2850 (2007).

3. Cheng, W.L. et al. Probing in Real Time the Soft Crystallization of DNA-Capped Nanoparticles. *Angew. Chem. Int. Ed.* **49**, 380-384 (2010).

4. Uetsuki, K. et al. Experimental Identification of Chemical Effects in Surface Enhanced Raman Scattering of 4-Aminothiophenol†. *J. Phys. Chem. C* **114**, 7515-7520 (2010).

5. Kim, K. & Yoon, J.K. Raman Scattering of 4-Aminobenzenethiol Sandwiched between Ag/Au Nanoparticle and Macroscopically Smooth Au Substrate. *J. Phys. Chem. B* **109**, 20731-20736 (2005).
